# Real-World Multicenter Evaluation of MEMOTIN® for Tinnitus in Private Clinic Settings: The M.E.S.T Study

**DOI:** 10.7759/cureus.91132

**Published:** 2025-08-27

**Authors:** Zacharias Kalentakis, Vasiliki Economou, Pavlos Maragkoudakis

**Affiliations:** 1 Otolaryngology - Head and Neck Surgery, Sismanoglio General Hospital of Attiki, Athens, GRC; 2 Biostatistics, Private Statistic Services, Athens, GRC; 3 Otorhinolaryngology, IASO General Clinic, Athens, GRC; 4 Otorhinolaryngology, National and Kapodistrian University of Athens, Athens, GRC

**Keywords:** management of tinnitus, otologic tinnitus, tinnitus annoyance, tinnitus handicap inventory, tinnitus improvement

## Abstract

There is currently no universally effective treatment for subjective tinnitus, and management strategies focus on the relief of symptoms rather than a definite cure. In line with this, our prospective, non-interventional, non-placebo-controlled, multicenter clinical study, conducted in private clinics, evaluates the efficacy of taking a daily supplement based on active compounds in alleviating tinnitus-related disability. The study was conducted in adults with tinnitus who were instructed to take one capsule daily, 30 minutes before bedtime, for a duration of three months between March 30 and December 20, 2024. This study aims to provide valuable clinical insights into the potential therapeutic benefits of a dietary supplement in alleviating the symptoms of tinnitus and improving patients’ quality of life. Employing validated assessment tools, such as the Tinnitus Handicap Inventory (THI), this study seeks to provide objective evidence regarding the potential benefits of MEMOTIN® as a supportive intervention for individuals suffering from tinnitus.

## Introduction

Tinnitus is a common auditory condition characterized by the perception of ringing, buzzing, or other phantom sounds without an external auditory stimulus, affecting approximately 10%-15% of the population [1]. Tinnitus is not a disease itself; however, it is often associated with hearing loss, exposure to noise, aging, or other underlying medical conditions and results in a variable degree of impact on the daily life of affected individuals [2]. For some individuals, tinnitus remains a mild, occasional annoyance; on the other hand, others experience a chronic and distressing condition, significantly impairing their sleep, concentration, mood, and overall well-being. 

Despite extensive research, there is currently no universally effective treatment for tinnitus, and management strategies focus on the relief of symptoms rather than a definite cure. Various therapeutic approaches, including sound therapy, cognitive behavioral therapy (CBT), pharmacological interventions (antidepressants: amitriptyline, trimipramine, and nortriptyline; anxiolytics: clonazepam, alprazolam, and diazepam; glutamate receptor antagonists: acamprosate, caroverine, memantine, etc.; and dietary supplements: *Ginkgo biloba*, vitamin B12, magnesium, zinc, and melatonin), have been explored to alleviate its burden [3-5].

The subjective character of tinnitus among individuals necessitates the evaluation of its impact on quality of life through validated patient-reported outcome measures (PROMs). These standardized questionnaires provide valuable insights into the psychological, functional, and emotional burden of tinnitus, as well as the effectiveness of interventions. One of the most widely used instruments for the assessment of tinnitus is the Tinnitus Handicap Inventory (THI) [6], a 25-item questionnaire designed to quantify the degree to which tinnitus affects a patient's daily life. The THI is divided into three subclasses: the functional subscale, which measures difficulties in concentration, hearing, and daily activities; the emotional subscale, which evaluates frustration, stress, and emotional distress associated with tinnitus; and the catastrophic subscale, which assesses extreme reactions such as feelings of helplessness or loss of control.

In addition to THI, other measures, such as sleep quality assessments, tinnitus severity scales, and treatment compliance tracking, are crucial in evaluating the effectiveness of interventions. Given the high prevalence of tinnitus-related insomnia, sleep disturbances are often a key factor in determining the overall success of treatment [7,8].

This study aims to provide valuable clinical insights into the potential therapeutic benefits of a dietary supplement in alleviating the symptoms of tinnitus and improving patients’ quality of life. Employing validated assessment tools, such as the THI, this study seeks to provide objective evidence regarding the potential benefits of MEMOTIN® as a supportive intervention for individuals suffering from tinnitus.

## Materials and methods

Study design

This study is a prospective, non-interventional, non-placebo-controlled, multicenter clinical study conducted in private clinics. The study aims to evaluate the effectiveness and safety of MEMOTIN®, a dietary supplement formulated for individuals with subjective tinnitus. The study was carried out following the tenets and principles of the Declaration of Helsinki and the Good Clinical Practice guidelines of the International Council for Harmonization. Informed consent was provided by all participants before their inclusion in the study.

Study product

The product under investigation is MEMOTIN®, which contains the following active ingredients per capsule: *Ginkgo biloba* (60 mg), magnesium (220 mg), zinc (10 mg), vitamin B12 (0.0025 mg), and melatonin (1.9 mg). Each component plays a distinct role in supporting neurological function, circulatory health, and sleep regulation, which are essential factors in the management of tinnitus [1,9-13] (Table 1).

**Table 1:** Memotin components and action Source: [1,9-13].

Component	Action
Ginkgo biloba	May improve blood flow to the inner ear and reduce oxidative stress by altering the tone of blood vessels [1,9]
Magnesium	Prevents cochlear damage from noise-induced hearing loss by reducing calcium influx into hair cells [1,10]
Melatonin	Known for its sleep-regulating properties, engagement with the sleep-wake cycle, and antioxidant activity [1,11]
Zinc	Supports and protects the cochlea from free radical damage, maintaining cochlear integrity, modulating neurotransmission, and exhibiting antidepressant effects [1,12]
Vitamin B12	Supports nerve function and may be beneficial for tinnitus linked to B12 deficiency [1,13]

Study visits and assessments

Patients were instructed to take one capsule daily, 30 minutes before bedtime, for a duration of three months. No additional recommendations regarding dietary or exercise regimen modifications were provided during the study. The study was scheduled across two visits. At the baseline (day 0) visit, patients were informed of the study’s objectives, procedures, and potential risks. Informed consent was obtained prior to the initiation of any study-related activities. Demographic information, medical history, and relevant baseline characteristics were collected. Patients then completed the THI questionnaire, a validated instrument designed to evaluate the impact of tinnitus on daily functioning and quality of life. At the 90-day follow-up (day 90), patients will again complete the THI questionnaire to assess any changes in tinnitus severity and its effect on the quality of life. Treatment adherence and compliance will be reviewed, and any reported adverse events (AEs) or side effects will be thoroughly documented.

Study population

This study was conducted in private clinics across Greece, including Athens, Thessaloniki, Chalkida, Veria, and Kavala, between March 30 and December 20, 2024.

Inclusion criteria

The study included adult patients diagnosed with subjective tinnitus lasting for more than six months. Participants were eligible if they were 18 years of age or older and had experienced tinnitus for over six months without having received any prior treatment for the condition. Additional inclusion criteria required participants to demonstrate adequate proficiency in the Greek language, as assessed by the validated Greek version of the THI. Furthermore, participants had to express their willingness to attend follow-up visits as required. All participants provided written informed consent, allowing their data to be collected and used for research purposes.

Exclusion criteria

Patients meeting any of the following criteria were excluded from the study: use of similar tinnitus treatments within the past three months, current treatment with anticoagulant medications, history of alcohol or substance abuse within the last six months, presence of serious otologic conditions (including Ménière’s disease, otosclerosis, otitis media, and/or eardrum perforation), diagnosed psychiatric disorders (including depression, psychosis, or other severe mental health conditions), pregnant or lactating women, patients with renal failure, known hypersensitivity or allergy to any of the supplement's ingredients, and patients with scheduled surgery during the study period.

Every exclusion criterion was based on documented medical histories and assessments during screening, although the specific methods used for ruling out these conditions (e.g., imaging, psychiatric evaluations) are not detailed, representing a possible methodological gap.

Study objectives

The primary objective was to assess the effectiveness of MEMOTIN® in reducing the impact of tinnitus on patients’ quality of life over a three-month treatment period. Changes in the THI total score and emotional subscale from baseline to three months were used as primary endpoints. Moreover, an evaluation of the safety and tolerability of MEMOTIN® during the study period was secondarily performed.

Assessment tool: Greek-validated version of the THI score 

The THI questionnaire is a widely recognized and validated instrument for quantifying the impact of tinnitus on an individual's daily life [6,14,15]. It consists of 25 items, categorized into three subscales: the functional subscale (11 items), which evaluates the impact of tinnitus on daily activities, including concentration and sleep; the emotional subscale (9 items), which assesses tinnitus-related psychological distress, such as frustration, anxiety, and depression; and the catastrophic subscale (5 items), which measures extreme negative reactions to tinnitus, such as feelings of helplessness or loss of control. In this study, the validated Greek translation of the THI questionnaire was utilized [15].

In conclusion, effectiveness outcomes were analyzed in the per-protocol (PP) population, which included 161 patients. The PP population consisted of all participants who did not have any major protocol deviations. Furthermore, safety data were analyzed in the safety population, which included 172 patients who received at least one dose of the treatment. Effectiveness outcomes are expressed as means ± standard deviation (SD) and were compared between visits using paired statistical tests. A p-value of less than 0.05 was considered statistically significant. The treatment was deemed effective if the THI score was reduced by at least 20%.

Data analysis

Descriptive statistics were presented as means ± standard deviation (SD) and used to summarize the distribution of demographic and clinical variables. Changes in outcome measures between baseline and follow-up visits were analyzed using the paired t-test for continuous variables. A p-value of less than 0.05 was considered statistically significant. Treatment effectiveness was defined as a ≥20% reduction in the total THI score compared to the baseline value, indicating a clinically meaningful improvement in tinnitus-related disability. Statistical analyses were performed using Python libraries (including pandas, seaborn, pingouin, and statsmodels) (Python Software Foundation, Fredericksburg, VA, USA). Using Python 3.13 along with standard scientific libraries, we performed paired t-tests to compare pre- and post-treatment scores. Subgroup analyses were also conducted to investigate potential differences in treatment response based on specific variables such as age, gender, and the presence of insomnia.

This study was approved by the Institutional Review Board of the IASO General Clinic. All data were encrypted before further analysis.

## Results

Study population

A total of 174 patients were included, of whom 161 (92.5%) completed the study. Two patients were lost to follow-up, nine patients had symptoms for less than six months, and two patients received the product for less than a month (Table 2).

**Table 2:** Demographics of the participants

Demographics	Ν = 161
Age	58.17 years (SD ± 12.64)
Age of tinnitus onset	55 years (SD ± 11.12)
Sex	
Female	77 (47.8%)
Male	84 (52.2%)
Hearing loss	
Yes	68 (42.2%)
No	93 (57.8%)

Reduction in total THI score

Following the administration of MEMOTIN®, the mean THI total score significantly decreased from 46.67 ± 21.33 at baseline to 25.11 ± 14.49 after 90 days of treatment. This corresponds to a mean reduction of 21.56 points (p = 0.0001), which exceeds both the clinically significant threshold of 7 points and the 20% reduction criterion (9.33 points relative to baseline) (Table 3). Out of 161 patients, 128 (about 80.1%) likely achieved a ≥20% reduction in THI score.

**Table 3:** Reduction in total THI score Python was used to perform paired t-tests to analyze differences between visits. A p-value of less than 0.05 was considered statistically significant. THI: Tinnitus Handicap Inventory.

Parameters	Results
Initial, mean ± SD	46.67 ± 21.33
Final, mean ± SD	25.11 ± 14.49
Difference	-21.56
% of reduction	46.19%
p-value	0.0001

THI emotional subscale

A statistically significant reduction was observed in the THI emotional subscale score, which decreased from 14.83 ± 8.21 at baseline to 8.30 ± 5.73 after treatment (p = 0.0001). This suggests a meaningful improvement in patients’ emotional responses to tinnitus (Table 4).

**Table 4:** THI emotional subscale score Python was used to perform paired t-tests to analyze differences between visits. A p-value of less than 0.05 was considered statistically significant. THI: Tinnitus Handicap Inventory.

Parameters	Results
Initial, mean ± SD	14.83 ± 8.21
Final, mean ± SD	8.30 ± 5.73
Difference	-6.53
% of reduction	44.03%
p-value	0.0001

Impact on insomnia

An analysis of insomnia-related responses showed that Category 2 (indicating that tinnitus sometimes affects sleep) was the most frequently reported category at baseline. Following the administration of MEMOTIN®, there was a statistically significant reduction in the impact of tinnitus on sleep (p < 0.05), demonstrating an improvement in sleep quality (Table 5).

**Table 5:** Treatment's impact on insomnia

Insomnia-related responses	Initial		Final	
	Frequency	(%)	Frequency	(%)
0	35	21.7	83	51.6
2	66	41.0	67	41.6
4	60	37.3	10	6.2
Missing values	0	0	1	0.6

Tinnitus severity scale

A descriptive analysis of the Tinnitus severity scale indicated a statistically significant reduction in tinnitus severity following the MEMOTIN® treatment (p < 0.05). This further supports the effectiveness of the supplement in alleviating the symptoms of tinnitus (Table 6).

**Table 6:** Treatment's impact on tinnitus severity scale

Tinnitus severity scale	Initial		Final	
	Frequency	(%)	Frequency	(%)
1	15	9.3	45	28.0
2	41	25.5	84	52.2
3	48	29.8	27	16.8
4	45	28.0	3	1.9
5	12	7.5	1	0.6
Missing values	0	0	1	0.6

Comparison of THI score with hearing loss and age

While 41% of participants had hearing loss, the purpose of this study was not to specify specific characteristics of hearing impairment (bilateral, unilateral, or the type (e.g., sensorineural)). However, a subgroup analysis was performed comparing tinnitus improvement between participants with and without hearing loss. A comparison between hearing loss and THI score improvement revealed no statistically significant association (p = 0.637). This suggests that pre-existing hearing loss does not influence the degree of improvement in THI scores; thus, MEMOTIN®'s effect was independent of hearing status. Similarly, a comparison between age and THI score improvement showed no statistically significant difference (p = 0.694), indicating that age does not affect the extent of improvement in tinnitus symptoms.

Patient compliance and safety

**Figure 1:**
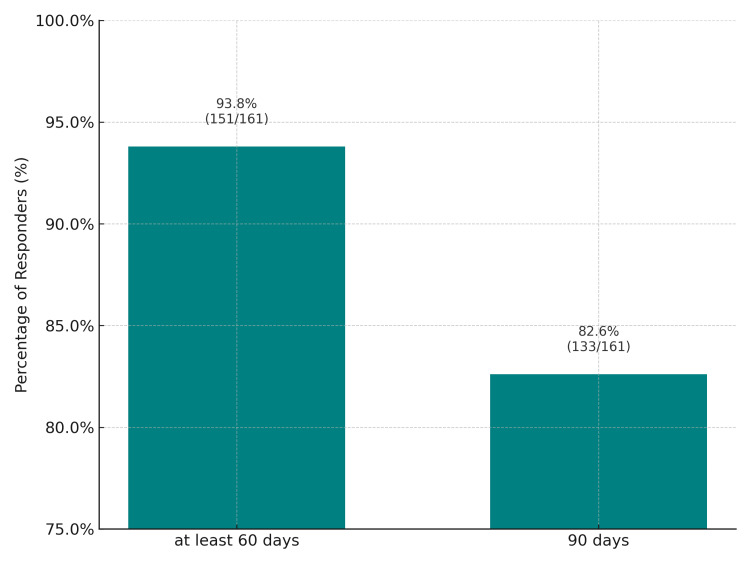
Patient's adherence: percentage of patients who completed at least 60 days vs. the full 90-day regimen

Patient adherence to treatment was high, with most participants completing the 90-day treatment period. A graphical representation illustrates the percentage of patients who completed at least 60 days versus those who completed the full 90-day regimen (Figure 1). Regarding safety, a total of four AEs were reported (2.3%), all of which were mild in intensity. These included one case each of headache, dizziness, overstimulation, and gastric discomfort. No participants discontinued the study due to these adverse events. In cases of early discontinuation, only patient-related factors lead to loss to follow-up.

## Discussion

Tinnitus remains a complex condition with no universally accepted pharmacological treatment, leading researchers to explore alternative therapeutic strategies, including dietary supplements. The potential of MEMOTIN®, a supplement containing *Ginkgo biloba*, magnesium, zinc, vitamin B12, and melatonin, to alleviate the symptoms of tinnitus was evaluated in this study. The combination of these ingredients addresses vascular health, neuronal protection, and sleep regulation, all of which are crucial factors in the pathophysiology of tinnitus.

While dietary supplements are often considered a complementary approach, they may offer a non-invasive and well-tolerated alternative for individuals seeking relief from tinnitus-related distress through improved emotional affectation while also maintaining a good safety profile [16]. The results of this study provide evidence that MEMOTIN® significantly reduced the severity of tinnitus and improved quality of life, particularly in terms of the THI total and emotional subscale scores. However, despite these promising findings, further research with larger sample sizes and long-term follow-ups is necessary to establish the consistency and reproducibility of these effects in broader populations.

Reduction in THI score: clinical relevance and interpretation

A key outcome of this study was the significant reduction in the THI total score following 90 days of MEMOTIN® supplementation. The mean THI score decreased by 21.56 points, exceeding the clinically meaningful threshold of 7 points and the 20% improvement criterion (i.e., 9.33 points relative to baseline). A prospective, interventional study with a similar dietary supplement showed a decrease in THI score by 15.7 points [16], whereas Cima et al. [17] presented a mean reduction of 22.3 points in THI scores among individuals who were treated according to the tenets of CBT, with this improvement lasting for 12 months.

The observed reduction suggests that MEMOTIN® effectively alleviates both the perceived intensity of tinnitus and its impact on daily life. More notably, the significant improvement in the THI emotional subscale (p = 0.0001) highlights its potential role in reducing tinnitus-related emotional distress. Emotional burden is a critical factor influencing patients’ well-being, often leading to frustration, anxiety, and, in some cases, depression. The observed improvements indicate that MEMOTIN® may not only reduce the perception of tinnitus but can also enhance emotional resilience and coping mechanisms. Interestingly, similar studies have provided evidence that tricyclic antidepressants (e.g., nortriptyline) can reduce emotional distress, especially in patients with comorbid depression, while other studies have reported a low grade of quality of life as a result of the failure to separate the effects of tinnitus from those of anxiety and depression. In contrast, selective serotonin reuptake inhibitors (SSRIs) may not provide overall improvements in any of the validated tinnitus-related parameters, except for a possible benefit associated with higher doses of the drug [18]. However, in any case of antidepressant therapy, the effects on the severity of tinnitus were limited. A notable reduction in emotional distress, including anxiety and catastrophic thinking, was also observed among patients with chronic tinnitus who underwent mindfulness-based cognitive therapy [19].

Impact on quality of life: sleep and psychological well-being

Tinnitus can concurrently coexist with other conditions such as anxiety, depression, dysfunction of the temporomandibular joint, hyperacusis and headache, difficulty concentrating, sleep disturbances, and even emotional distress [20], resulting in a severely impacted quality of life. Wang et al. [21] found that the mediating effect of sleep disorders between the severity of tinnitus and anxiety accounted for 27.88% of the total effect size. In this study, insomnia-related complaints showed a statistically significant reduction (p < 0.05), indicating that MEMOTIN® may help mitigate sleep disturbances frequently associated with tinnitus. Given that melatonin is a key component of MEMOTIN®, its well-documented role in sleep regulation and antioxidant protection may contribute to this positive outcome.

Furthermore, improvements in tinnitus severity scores (p < 0.05) reinforce the potential benefits of MEMOTIN® in reducing tinnitus-related distress. However, it is important to recognize inter-individual variability, as the perception of tinnitus and responses to treatment can be influenced by psychological factors, lifestyle, and underlying medical conditions. The findings of our study suggest that incorporating dietary supplementation as part of a holistic tinnitus management plan may provide meaningful relief for certain individuals.

The value of a multicenter clinical study in private clinics

One of the strengths of this study is its rigorous multicenter design, which increases the generalizability of the results. Conducting the study in private clinics provided several advantages: firstly, a real-world clinical setting, where patients seek treatment under normal healthcare conditions; a diverse patient population, improving the external validity of the findings; and finally, an enhanced follow-up compliance, as private healthcare settings often foster stronger patient-practitioner relationships.

Additionally, centralized data collection and validation across multiple sites reduce biases and enhance the credibility of the results. While randomized controlled trials (RCTs) remain the gold standard for clinical research, real-world studies conducted in private practice settings offer valuable insights into patient adherence, treatment efficacy, and tolerability in everyday scenarios. Nonetheless, a carefully conducted multicenter study with systematic data collection and strong statistical analysis can yield clinically significant and widely applicable results. Various observational study designs may be utilized, such as cross-sectional, cohort, and case-control approaches [22].

Comparison with previous studies

The results of this study align with the findings of previous research investigating the use of dietary supplements for tinnitus management. *Ginkgo biloba*, one of the key components of MEMOTIN®, has been studied for its potential role in improving circulatory function and reducing oxidative stress in the auditory system. Some studies have reported modest improvements in the severity of tinnitus, while others have suggested no significant benefit compared to placebo. Clearly, the available literature shows mixed results [1]. Both Krauss et al. [23] and von Boetticher [24] highlighted the beneficial outcomes of *Ginkgo biloba*, while a review of 12 studies including 1,915 participants carried out by Sereda et al. [5] showed that *Ginkgo biloba* may have little to no effect on THI scores at three to six months compared to placebo.

Additionally, melatonin, a naturally occurring indoleamine that helps regulate circadian and circannual rhythms, has been previously studied for its effects on tinnitus-related sleep disturbances, with positive findings indicating its role in reducing the perception of tinnitus, improving sleep quality, and enhancing overall well-being. Based on research findings, melatonin has been demonstrated to have a protective role against cochlear damage caused by acoustic trauma and ototoxic substances, while clinical studies have shown that it can reduce the severity of tinnitus symptoms. As a result, melatonin is considered a potentially beneficial treatment option for individuals with tinnitus, particularly considering its influence on neural plasticity, oxidative and nitrosative stress, apoptosis, and autophagy [25]. Our study supports these earlier findings by demonstrating significant improvements in THI scores and sleep-related distress.

However, not all studies investigating dietary supplements for tinnitus have yielded consistent results. Variability in study designs, dosages, treatment durations, and patient populations may contribute to these discrepancies. Compared to previous trials, the present study demonstrated a larger and statistically significant reduction in THI scores, reinforcing the potential role of MEMOTIN® as a supportive intervention for tinnitus sufferers.

Indeed, our study was a non-placebo-controlled, non-interventional real-world study, which limits the ability to distinguish the true efficacy of MEMOTIN® from placebo effects. The current study's real-world design was intended to assess MEMOTIN® in typical clinical settings, emphasizing feasibility and tolerability, but this naturally reduces internal validity regarding causality. Future research should focus on larger, placebo-RCTs to confirm these findings. More long-term follow-up studies are necessary to assess the sustainability of the observed improvements. Comparative studies evaluating MEMOTIN® against other tinnitus management strategies, including sound therapy, behavioral interventions, and pharmacological options, should also be carried out.

Limitations

This study has several limitations that should be acknowledged. First, the study was not registered in a clinical trials database, which is a limitation. It was started as an exploratory pilot investigation, and registration was not initially planned. The absence of a control group limits the ability to definitively attribute observed improvements to MEMOTIN®, as the placebo effect, commonly seen in tinnitus studies, cannot be ruled out. Additionally, the open-label design may have introduced expectation bias, potentially influencing both patient-reported outcomes and clinician assessments. The setting of private clinics may have led to selection bias, as participants may differ from the general population in terms of socioeconomic status, healthcare access, or health-seeking behavior, thereby affecting the generalizability of the findings. The study relied on subjective outcome measures, such as self-reported questionnaires (e.g., THI), without the inclusion of comorbidities and objective assessments like audiometry, blood tests, or neuroimaging. Moreover, the follow-up period may have been too short to evaluate the long-term effectiveness and safety of MEMOTIN®, particularly given the chronic nature of tinnitus. Uncontrolled confounding variables, such as lifestyle changes, concurrent treatments, or variations in sleep and stress levels, may also have influenced the results. Finally, as a real-world multicenter study, there was likely variability in clinical practice, including differences in protocol adherence and patient monitoring, which could have impacted the consistency of the data collected.

## Conclusions

This study provided compelling evidence that MEMOTIN® supplementation may lead to statistically and clinically significant improvements in the severity of tinnitus, the associated emotional burden, and sleep quality. These results highlight the potential role of dietary supplements as an adjunctive therapy for managing tinnitus. Moreover, the multicenter private clinic-based study design enhances the validity and applicability of the findings regarding real-world clinical practice. While the findings are promising, further research through larger RCTs with extended follow-up is warranted to establish MEMOTIN® as a reliable and standardized approach for tinnitus relief. Such results will contribute to understanding the supplement’s efficacy and safety profile, offering guidance for future clinical applications.
